# Diversity in Neuroanatomical Distribution of Abnormal Prion Protein in Atypical Scrapie

**DOI:** 10.1371/journal.ppat.0030082

**Published:** 2007-06-08

**Authors:** Alice Nentwig, Anna Oevermann, Dagmar Heim, Catherine Botteron, Karola Zellweger, Cord Drögemüller, Andreas Zurbriggen, Torsten Seuberlich

**Affiliations:** 1 NeuroCenter, Reference Laboratory for TSE in Animals, Vetsuisse Faculty, University of Berne, Berne, Switzerland; 2 Federal Veterinary Office, Liebefeld, Switzerland; 3 Institute of Genetics, Vetsuisse Faculty, University of Berne, Berne, Switzerland; University of Toronto, Canada

## Abstract

Scrapie is a transmissible spongiform encephalopathy (TSE) in sheep and goats. In recent years, atypical scrapie cases were identified that differed from classical scrapie in the molecular characteristics of the disease-associated pathological prion protein (PrP^sc^). In this study, we analyze the molecular and neuropathological phenotype of nine Swiss TSE cases in sheep and goats. One sheep was identified as classical scrapie, whereas six sheep, as well as two goats, were classified as atypical scrapie. The latter revealed a uniform electrophoretic mobility pattern of the proteinase K–resistant core fragment of PrP^sc^ distinct from classical scrapie regardless of the genotype, the species, and the neuroanatomical structure. Remarkably different types of neuroanatomical PrP^sc^ distribution were observed in atypical scrapie cases by both western immunoblotting and immunohistochemistry. Our findings indicate that the biodiversity in atypical scrapie is larger than expected and thus impacts on current sampling and testing strategies in small ruminant TSE surveillance.

## Author Summary

In the view of concerns that bovine spongiform encephalopathy has entered the small ruminant population, comprehensive active surveillance programs for transmissible spongiform encephalopathies (TSEs) in sheep and goats were implemented worldwide. In these, previously unrecognized atypical scrapie cases were identified that to date represent the majority of detected small ruminant TSE cases in some countries. The pathogenesis and epidemiology of atypical scrapie, as well as its relevance to both animal health and food safety, is still poorly understood. In the present study, we performed a systematic neuropathological analysis of recently diagnosed atypical scrapie cases in Switzerland. Our results show that the neuropathological presentation in atypical scrapie–affected small ruminants varies remarkably, and the results indicate a biodiversity of TSEs in sheep and goats larger than expected, with some similarities to known human TSEs. These findings will form the basis for future research on TSE phenotypes and help to design experimental studies necessary to generate data for risk assessments and the implementation of appropriate disease-control strategies.

## Introduction

Transmissible spongiform encephalopathies (TSEs) are fatal neurodegenerative diseases that are caused by prions [1] and include, among others, Creutzfeldt-Jakob and Gerstmann-Sträussler-Scheinker disease (GSS) in humans, bovine spongiform encephalopathy (BSE) in cattle, and scrapie in small ruminants (SRs), comprising sheep and goats. A hallmark of TSEs is the deposition of a misfolded, partially proteinase K–resistant isoform (PrP^sc^) of the host-encoded normal prion protein (PrP^c^). Current molecular diagnostic procedures rely on the detection of the proteinase K–resistant core fragment, termed PrP^res^. Concerns were raised that BSE might have entered the SR population unnoticed. Moreover, in experimental SR BSE, infectivity was detected in various nonnervous tissues [2] and body fluids [3] and may present a risk for consumers and livestock. As a consequence, in 2002, European Union member states enhanced TSE surveillance in SRs, and subsequently, the first natural BSE case in a French goat was identified [4]. Besides this, numerous so-called atypical scrapie cases that differed in their neuropathological and molecular characteristics from classical scrapie and experimental SR BSE were first reported in Norway [5] and later in other countries (reviewed by [6]). Importantly, such cases also occurred in sheep with genotypes considered to confer resistance to scrapie and were missed by some screening test formats while others readily detected them [7]. In the meantime, specific SR TSE screening tests have been evaluated and approved by the authorities. Consequently, the number of atypical scrapie cases reported increased, but still very little information is available about the phenotype and epidemiology of this disease. To date, three forms of SR TSE can be discriminated by molecular PrP^res^ typing [8]: classical scrapie, atypical scrapie, and BSE.

In the present study, we aimed at analyzing the complete set of SR TSE cases detected by active and passive surveillance in Switzerland from 2004 to 2005 for the molecular and neuropathological phenotype, and at assessing the efficacy of current testing strategies. Our results indicate unexpected variations in the phenotype of atypical scrapie in sheep and goats.

## Results

### Confirmation of SR TSE

In the framework of a 1-y active surveillance program, more than 30,000 regularly slaughtered (RS) and more than 3,000 fallen SR stock (FS) were analyzed for TSE in Switzerland. RS animals were screened with either of the two approved screening tests: the Prionics Check Western SR (referred to as test A) and the Bio-Rad TeSeE sheep and goat ELISA (referred to as test B) in caudal brainstem samples. By contrast, FS, as well as clinically suspicious (CS) animals were analyzed by immunohistochemistry (IHC) in different brain structures and both screening tests in caudal brainstem samples in parallel. Applying this testing scheme, nine SR TSE cases (Table 1) were identified and confirmed by IHC. In three of these animals, clinical signs of a central nervous system disorder (Table 1) were reported. Lymphoid tissues were available in four cases (S5/FS, S6/FS, S7/CS, and G2/FS) and tested negative with screening test B (retropharyngeal lymph node) and by IHC (retropharyngeal lymph node, tonsils).

**Table 1:**
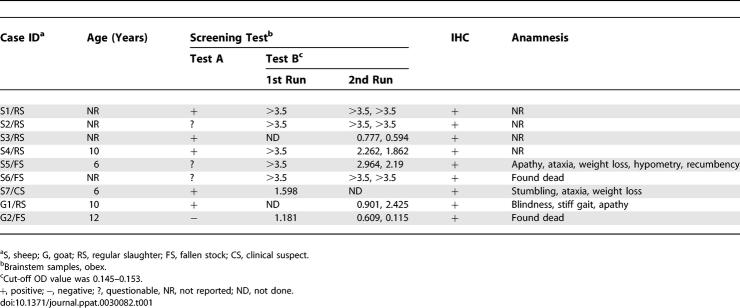
Results of Screening Tests, IHC, and Anamnesis of the TSE Cases in SRs Identified in Switzerland 2004–2005

### TSE Screening Test Results on Brainstem Samples

**Figure 1:**
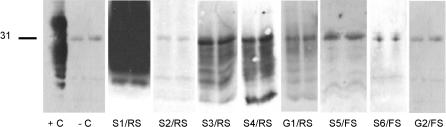
Initial Results of Confirmed TSE Cases in Screening Test A, a WB Procedure Brainstem samples were analyzed in duplicate for the presence of PrP^res^. The band at 31 kDa (as indicated on the left) results from an unspecific binding of the secondary antibody to proteinase K. Case ID numbers are given at the bottom. +C, test-kit control, undigested brain tissue. −C, negative control sample from a negative-confirmed sheep.

In slaughtered SRs, ~47% of the animals were screened with test A and ~53% with test B in regional laboratories. Two slaughtered TSE cases were initially picked up by test A (S3/RS and G1/RS) and three by test B (S1/RS, S2/RS, and S4/RS). The respective screening tests were repeated in the reference laboratory, and fresh brainstem samples from the same animals were cross-checked with the second screening test. All nine TSE cases scored positive in test B, but large variations in the optical density values were observed between replicates for some samples (Table 1). By contrast, only five TSE cases gave a clear positive signal in test A (Figure 1 and Table 1).

### Molecular PrP^res^ Typing

**Figure 2:**
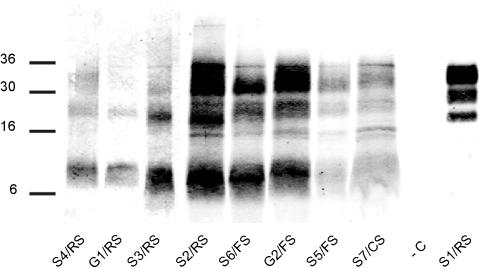
Comparison of PrP^res^ in SR TSE Cases by Refined WB Analyses were performed on brainstem samples (S1/RS–S4/RS, −C). In animals with weak positive signals in brainstem samples, tissue from cerebellar cortex (S5/FS–S7/CS, G1/RS) or basal ganglia (G2/FS) were used. A TSE-negative sheep sample served as negative control. Molecular mass standards in kDa are indicated on the left. −C, negative control sample.

**Figure 3:**
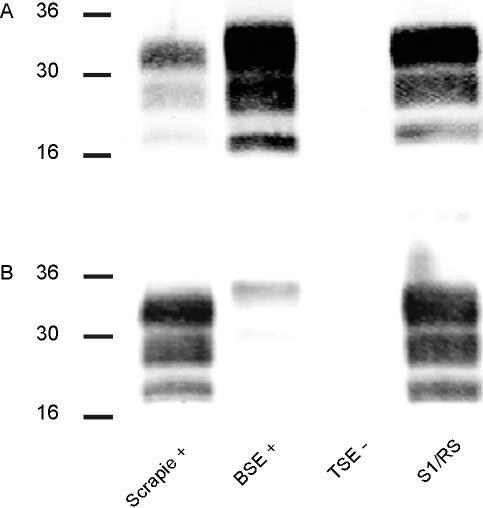
Discriminatory WB of Case S1/RS Brain tissue homogenates were digested with proteinase K and PrP^res^ detected with MAb 6H4 (A) and P4 (B). A BSE-positive, a scrapie-positive, and a scrapie-negative sample served as controls. Molecular mass standards in kDa are indicated on the left.

**Figure 4:**
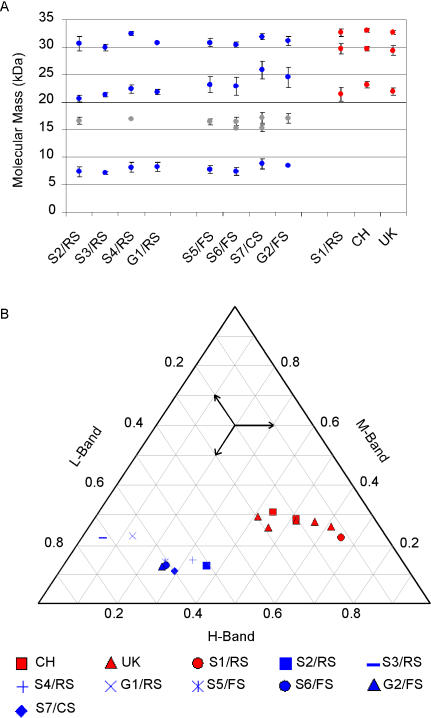
EMPs of PrP^res^ in Atypical (Blue) and Classical (Red) Scrapie Cases Swiss SR TSE cases compared to classical scrapie reference cases from Switzerland (CH) and the United Kingdom (UK) in WB. (A) Molecular masses of individual PrP^res^ bands. Major bands are displayed with blue or red circles, minor bands with gray circles. Mean values and standard deviations (ι) were calculated from at least five individual test runs. (B) Triangular plot of the proportions of the three major PrP^res^ bands (H: high, M: mid, L: low molecular mass); minor bands were not included in the plot. Arrows indicate how to read the plot.

For further classification of the TSE cases identified, a refined western immunoblot (WB) method was used, resulting in an improved resolution of individual PrP^res^ bands. Analyses were undertaken repeatedly, and molecular masses and relative signal intensities of PrP^res^ bands were measured. Case S1/RS showed a three-band pattern, with moieties migrating at ~33 kDa, ~30 kDa, and ~21 kDa, respectively (Figure 2). This electrophoretic mobility pattern (EMP) is indicative for either classical scrapie or BSE [8]. Consequently, the sample was analyzed using a discriminatory WB technique, thereby presenting features of classical scrapie and not of BSE (Figure 3). All other SR TSE cases revealed a different multiband pattern with three major bands migrating at a molecular mass of ~8 kDa, ~23 kDa, and ~31 kDa, respectively (Figure 2). In animals with high PrP^res^ signal intensities, one to two additional minor bands in the range of 15–17 kDa were observed. Based on these observations and according to defined criteria [8], these TSE cases were classified as atypical scrapie. All atypical scrapie cases revealed a consistent EMP (Figure 4A) and similar proportions of the intensity of bands (Figure 4B), although some slight variations between individual samples were observed. With both criteria, atypical scrapie cases clearly differed from Swiss and British classical scrapie reference cases and S1/RS.

### PrP^res^ Distribution Profiling by WB

**Figure 5:**
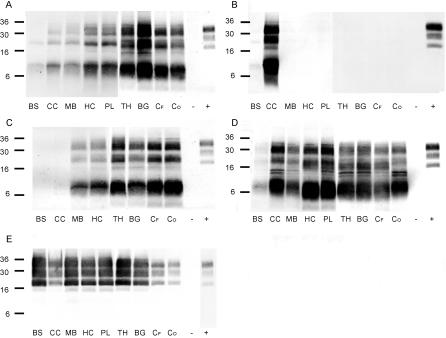
PrP^res^ Distribution Profiles in Brains of Atypical and Classical Scrapie–Affected SRs by WB Atypical scrapie cases S5/FS (A), S6/FS (B), and S7/CS (D) in sheep and G2/FS (C) in a goat compared to a classical scrapie reference case from Switzerland (E). For comparison between individual membranes, the same amount of a classical scrapie sample (+) was loaded onto each gel. Neuroanatomical structures: BS, caudal brainstem; CC, cerebellar cortex; MB, midbrain; HC, hippocampus; PL, piriform lobe; TH, thalamus; BG, basal ganglia; C_F_, frontal cortex; C_O_, occipital cortex. TSE-negative brain tissue served as negative control (−).

In four of the atypical scrapie cases (S5/FS, S6/FS, S7/CS, and G2/FS) whole brains were available. In order to estimate the distribution profile of abnormal prion protein throughout the brains of these animals, samples of representative neuroanatomical structures were analyzed by the refined WB and compared to those of Swiss and British classical scrapie cases. Very similar EMPs were observed in all four atypical cases in these structures (Figure 5A–5D). In G2/FS (Figure 5C), PrP^res^ was not detected in the caudal brainstem and the cerebellar cortex. In all other structures examined, especially in thalamus, the basal ganglia, and the cerebral cortex, it was present in high levels. Interestingly, a completely diverse picture was observed in sheep S6/FS where PrP^res^ was detected in the cerebellar cortex, and barely in caudal brainstem, but not in the more rostral structures (Figure 5B). In sheep S7/CS (Figure 5D) and S5/FS (Figure 5A), PrP^res^ deposits were most pronounced in the cerebellar cortex, hippocampus, basal ganglia, and the piriform lobe, but again the caudal brainstem sample reacted weakly. By contrast, in the classical scrapie reference cases from the United Kingdom (unpublished data) and Switzerland (Figure 5E), PrP^res^ accumulations were consistently detected at high amounts, notably in the caudal brainstem, but also in all other structures examined.

### PrP^sc^ Distribution Profiling by IHC

**Figure 6:**
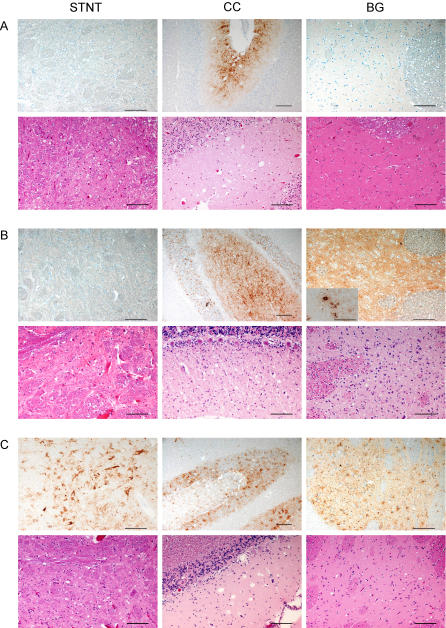
IHC and Histopathology of Selected Brain Structures in Atypical and Classical Scrapie–Affected SRs Comparison of PrP^sc^ immunolabeling with monoclonal antibody F99/97.6.1 in IHC (upper panel) and vacuolar lesion in histopathology (lower panel) in atypical scrapie cases S6/FS (A) and S7/CS (B) in sheep, compared to a classical scrapie reference case from Switzerland (C) in different neuroanatomical brain structures: brainstem, spinal tract nucleus of the trigeminal nerve (STNT); cerebellar cortex (CC), and basal ganglia (BG). (B) The inset for S7/CS, basal ganglia, shows plaque-like PrP^sc^ deposits in the ventral and the medial geniculate body of the thalamus. Bars indicate 100 μm, except for the IHC of CC (150 μm).

Next, we attempted to specify the findings in WB by using an alternative prion protein (PrP) detection method. To this end, available formalin-fixed brain tissues of all SR TSE cases identified and a Swiss classical scrapie reference case, were analyzed by IHC, and the type and intensities of PrP^sc^ deposits in defined neuroanatomical structures were assessed by light microscopy (Table 2). In the atypical scrapie cases, the PrP^sc^ deposition type consisted of fine to coarse granules that were found in the gray and white matter (Figure 6A and 6B). All the atypical cases, except S6/FS, revealed a faint positive immunolabeling in the caudal brainstem that involved mainly the spinal tract nucleus of the trigeminal nerve, and in two cases (S7/CS and S5/FS) additionally the reticular formation and the ambiguus nucleus. The dorsal motor nucleus of the vagus nerve was not affected. One remarkable finding was that cases S7/CS and S5/FS had equal amounts of PrP^sc^ in the molecular and the granular layer of the cerebellar cortex, whereas cases S2/RS and S3/RS showed severe accumulations in the molecular layer, but to a much lesser extent in the granular layer. In line with the findings in WB, cases S7/CS, S5/FS, and G2/FS showed moderate to severe amounts of PrP^sc^ in the more rostral structures, such as thalamus, basal ganglia, and cerebral cortex. Case S6/FS was essentially negative in all of these structures. It is noteworthy that in sheep S7/CS, dense, plaque-like deposits were identified in the ventral and the medial geniculate body of the thalamus (Figure 6B, inset).

**Table 2:**
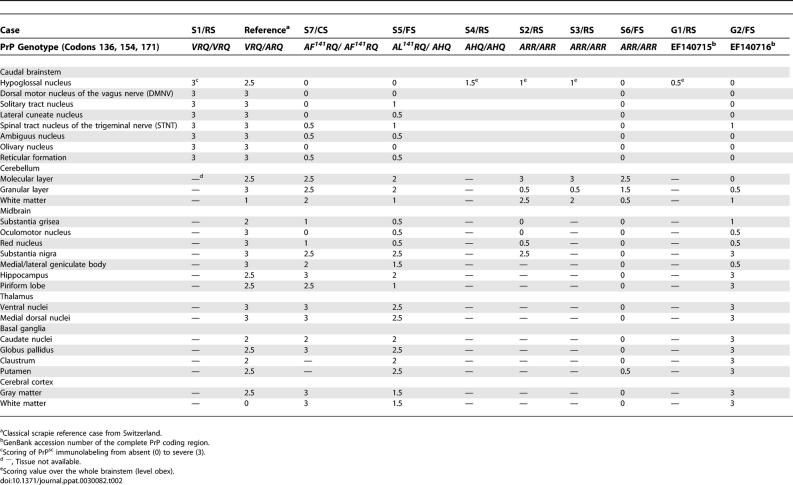
Anatomical Distribution and Severity of PrP^sc^ Accumulation in IHC

In the classical scrapie case, S1/RS, severe amounts of intraneuronal, perineuronal, and coarse granular type PrP^sc^ were detected in the caudal brainstem. In the Swiss classical scrapie reference case (Figure 6C), PrP^sc^ labeling of neurons and neuropil was detected throughout the brain in high amounts. Here the deposits were of the intraneuronal, perineuronal, and stellate, as well as coarse granular type, and involved also the dorsal motor nucleus of the vagus nerve of the caudal brainstem. Overall, the PrP^sc^ distribution profile of SR TSE cases as determined by IHC correlated well with the results of the refined WB analyses in all animals, with some minor variations.

### Histopathology

The interpretation of the haematoxylin and eosin–stained tissue slides of cases S1/RS, S2/RS, S3/RS, S4/RS, and G1/RS was not possible due to autolysis of the tissue and/or wrong tissue treatment such as freezing prior to embedment. Nevertheless, in cases S5/FS, S6/FS, S7/CS, and G2/FS spongiform lesions were identified in the neuropil that were generally associated with PrP^sc^ accumulation. In these four cases, spongiform lesions were absent in the brainstem at the level of the obex. In cases S7/CS (Figure 6B) and S5/FS, moderate to severe vacuolation was found in the molecular layer of the cerebellar cortex, the basal ganglia, and the cerebral cortex, whereas the midbrain and hippocampus were only mildly to moderately affected. Case S6/FS showed only mild vacuolation in the molecular layer of the cerebellar cortex (Figure 6A). Goat G2/FS has already been described elsewhere [9]. No intraneuronal vacuoles were observed in any of the structures examined. A diffuse gliosis appeared in cases S7/CS and G2/FS.

In the classical scrapie case, S1/RS, interpretation of vacuolation in the neuropil was complicated, but mild intraneuronal vacuolation was identified in the dorsal motor nucleus of the vagus nerve (unpublished data).

## Discussion

In this study, we demonstrate that the vast majority of SR TSE cases identified in Switzerland by active surveillance were atypical scrapie. Their PrP^res^ EMP was consistent, but prominent differences in the distribution of abnormal prion protein in the brains compared to each other and to those in classical scrapie–affected sheep were observed. Importantly, none of the cases revealed evidence for an infection with the BSE agent.

According to the literature, atypical scrapie is characterized by a PrP^res^ EMP with at least three to five bands, including a prominent lower band, estimated to migrate at 7–12 kDa [5,10–12]. In the present study, a detailed analysis of PrP^res^ in Swiss atypical scrapie cases showed a uniform multiband EMP with three major bands migrating at ~8 kDa, ~22 kDa, and ~32 kDa. In a previous study, a side-by-side comparison by WB showed that the PrP^res^ EMPs of sheep S7/CS, goat G2/FS, and a Nor98 case, the prototype of atypical scrapie, were essentially indistinguishable [9]. These results indicate that the Swiss atypical scrapie cases share a common molecular PrP^res^ type similar to Nor98 regardless of the host species and genotype.

The phenotype of a TSE is defined, among other criteria, by the neuroanatomical distribution of histopathological lesions and PrP^sc^ deposits. Most atypical scrapie cases were identified by active TSE surveillance; complete brain was usually not available because sampling is performed through the foramen magnum accessing the brainstem (and ideally also the cerebellum), but not the more rostral structures. Thus, until this date, very limited information is available about these criteria in brains of atypical scrapie–affected animals. In the literature for brainstem, no or only weak PrP^sc^ deposits were detected [5,7], whereas strong deposits were described for the cerebellum [5,13–15]. Positive WB signals have also been shown in samples from the cerebral cortex [5,15], midbrain [14], and thalamus [15].

In this study, we present for the first time a systematic comparative analysis of the PrP^sc^ distribution in brains of atypical scrapie–affected SRs by both WB and IHC. Our results show, that unlike some human TSEs [16], the PrP^res^ EMP in WB and the PrP^sc^ deposition type in IHC remains constant regardless of the brain structure examined. However, when we analyzed the neuroanatomical PrP^sc^ distribution by WB and IHC prominent differences were observed. In one goat (G2/FS), the cerebrum, thalamus, and midbrain were severely affected with a minimal involvement of the cerebellum and the caudal brainstem (Figure 5C and [9]). By contrast, a completely different pattern was observed in one sheep (S6/FS) where PrP^sc^ accumulated in the cerebellar cortex almost exclusively (Figures 5B and 6A). In other cases (S5/FS and S7/CS), severe deposits were detected in the cerebrum, thalamus, and midbrain and also involved the cerebellum to different extents, but again barely the caudal brainstem (Figure 5A and 5D). Small differences between IHC and WB results were observed but can be biased by the sampling procedure of frozen tissues for WB. Unfortunately, a complete and detailed analysis of the RS cases was not possible, due to the lack of whole brains and severe tissue autolysis. However, some brain structures, in addition to caudal brainstem, were available for IHC of S2/RS and S3/RS, and both cases seem to fit into either of the schemes found in S5/FS and S7/CS or S6/FS, respectively. The localization and severity of spongiform lesions in S5–S6/FS, S7/CS, and G2/FS correlated well with the PrP^sc^ deposits in IHC. Taken together, our results indicate a considerable diversity in the neuropathological representation of atypical scrapie in SRs.

A possible explanation for these findings is that the animals died at different time points in the course of the disease. One could speculate that PrP^sc^ deposits and histopathological lesions evolve from the cerebrum to the cerebellum, and to a lesser extent, the caudal brainstem during the pathogenesis. This concept may account for cases S5/FS and S7/CS, since both were clinically diseased with prominent signs of a central nervous system disorder. However, the PrP^sc^ distribution in S6/FS clearly cannot be explained by this means. Moreover, in S2/RS and S3/RS the cerebellum was severely affected, but these animals were regularly slaughtered, which implicates that clinical signs were not obvious at that time. Thus, the heterogeneities observed do not seem to reflect different stages of the disease exclusively. Another factor that may contribute to the phenotype of atypical scrapie is the PrP genotype of the host. Epidemiological studies indicate associations between the *AF^141^RQ* and the *AHQ* PrP alleles with atypical scrapie in sheep [17,18]. Only one of the Swiss atypical scrapie cases (S7/CS) encoded the *AF^141^RQ* allele, but three were homozygote for *ARR,* a genotype that is considered to confer resistance to classical scrapie. An interesting observation was that, in contrast to the other sheep, in *ARR/ARR* genotypes, the cerebellar cortex in the molecular layer was consistently affected in a much more severe manner compared to the granular layer. Based on our data we can not establish a clear correlation between the neuropathological diversity and the PrP genotype in scrapie-affected sheep. More field cases and data from experimental transmission studies are needed. Hence, the cause of neuropathological diversities in atypical scrapie cases remains unclear and may be linked to other host factors, the infectious agent involved, or the interaction of both.

**Figure 7:**
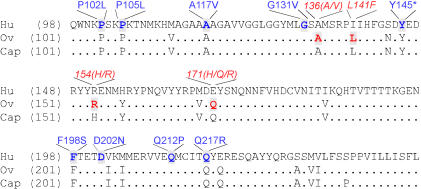
Partial Alignment of the PrP Amino Acid Sequences of Sheep, Goats, and Humans Mutations found in GSS are in blue, polymorphisms related to susceptibility for classical and atypical scrapie are indicated in red. Homologies between sequences are displayed with a dot. Hu, human; ov, ovine; and cap, caprine.

The molecular phenotype in atypical scrapie is unique for animal TSEs, but reminiscent to the one described for GSS. In GSS, also unique for human TSEs, a prominent PrP^res^ band similarly migrating at ~7 kDa in WB analysis has been described [19]. These lower fragments in atypical scrapie [10,11] and GSS [20] correspond to N- and C-terminal truncated PrP peptides. Since it is also detectable in nonproteinase K–treated tissue homogenates, it was suggested that these peptides are produced in vivo by a proteolytic pathway that partially degrades PrP in the extracellular compartment. PrP^sc^ deposition profiles very similar to G2/FS, S5/FS, S6/FS, and S7/CS have been described for GSS: a cerebellar type [21], a cerebral type, and combinations of both [22]. These distribution profiles are correlated to defined mutations in the host's PrP gene. To identify common genetic components in atypical scrapie and GSS, we compared the amino acid sequences of the prion protein in Swiss atypical scrapie cases with those found in GSS patients (Figure 7), but a common genetic background in the two diseases was not observed since the mutations described for GSS were absent in atypical or classical scrapie cases. Further, polymorphisms known to determine resistance or susceptibility to TSE in SRs were not found in GSS patients. Another striking similarity to GSS is the presence of plaque-like PrP^sc^ aggregations in the thalamus of S7/CS. Such PrP^sc^ deposits are a major feature of GSS in humans [22] and have not yet been described for atypical scrapie. None of the other cases under investigation showed this deposition type. It must be pointed out that S7/CS was in an advanced stage of the disease, and thus, at best comparable with samples derived from GSS patients. Whether these plaques are a general feature in late-stage atypical scrapie cases remains to be determined. For GSS the hereditary background is evident and an epidemiological link between these two diseases is not obvious until to date. However, since a direct comparison of GSS with atypical scrapie in the same experimental setup is still missing, one can only speculate that both diseases involve similar pathomolecular mechanisms. Atypical scrapie may therefore represent a valuable model for investigations in the pathogenesis of GSS and vice versa.

From the diagnostic point of view, our results were unexpected, as none of the structures examined was consistently affected in all of the animals. This has a strong impact on sampling techniques in terms of active and passive SR TSE surveillance. European Union member states have implemented sampling of cerebellum in addition to the standard brainstem sample after increasing reports of atypical scrapie. Our results sustain this action, insofar as the cerebellum indeed consistently revealed higher amounts of PrP^sc^ compared to caudal brainstem samples. For cases of a cerebellar type (like S6/FS) this procedure seems to be optimal. Beyond this, in most of the cases, especially in G2/FS, samples from the central parts of the brain such as thalamus or basal ganglia would be optimal to ensure a maximum in the sensitivity of PrP^sc^ detection. However, such a strategy requires sampling of the whole brain and should be possible in passive surveillance but may be unrealistic due to practical and economical constraints for large active surveillance programs in most countries. This dilemma may partially be overcome by the application of highly sensitive screening tests on cerebellum samples. It would be wise to reconsider current sampling and testing strategies and to enhance disease awareness in order to increase the efficacy of passive TSE surveillance schemes.

It has been demonstrated that atypical scrapie involves a transmissible agent [23], but as it mainly occurs as an isolated case in a flock, it remains unclear whether the disease can spread naturally in the SR population. Atypical scrapie is possibly a sporadic TSE in SRs. Next, our work will be extended to comparative transmission studies in rodent models and SRs for a further characterization of the infectious agents involved and the biodiversity in the phenotype of atypical scrapie.

## Materials and Methods

### TSE cases and reference material.

Eight TSE cases were detected via active surveillance in sheep (S1/RS–S6/FS) and goats (G1/RS and G2/FS) (Table 1). A clinically affected scrapie case (S7/CS) diagnosed in Switzerland in 2004 by passive TSE surveillance was included in this study. Classical scrapie reference material originated from two sheep inoculated with central nervous system material from a Swiss clinically scrapie–affected ram, and five British classical scrapie cases were provided from the Veterinary Laboratory Agency Archive, Weybridge, United Kingdom. BSE-positive control tissue was taken from a clinically affected Swiss cow. Negative control tissues were derived from sheep testing negative with both rapid and confirmatory tests.

### TSE screening tests.

In Switzerland, the Check Western SR (Prionics, http://www.prionics.com), referred to as screening test A, and the TeSeE Sheep/Goat ELISA (Bio-Rad, http://www.bio-rad.com), referred to as screening test B, were approved for TSE screening in SRs. Both tests were performed according to the manufacturer's instructions.

### Refined WB.

PrP^res^ was purified according to the protocol of the TeSeE Sheep/Goat Western Blot (Bio-Rad), separated by SDS PAGE, immunoblotted on nitrocellulose membranes, and detected with the monoclonal antibody P4 (R-Biopharm, 400 ng/ml; http://www.biopharm.com) and the secondary goat anti-mouse antibody IRDye 800 (200 ng/ml; Rockland, http://www.rockland-inc.com) using an Odyssey infrared imager (LI-COR Biosciences, http://www.licor.com). Protein bands were identified, quantified, and their molecular mass estimated with the Odyssey analysis software version 2.1 (LI-COR Biosciences).

### Discriminatory WB.

For the BSE/scrapie discriminatory WB, samples were processed as described for the refined WB, but PrP^res^ was detected separately with MAb P4 and MAb 6H4 (200 ng/ml; Prionics) in parallel.

### IHC and histopathology.

For histopathology, 4-μm-thick sections were deparaffinized and stained with haematoxylin and eosin. Confirmatory IHC and the PrP^sc^ distribution profiling in case G2/FS was performed as described previously [9]. For the PrP^sc^ distribution profiling in sheep, the protocol was optimized as follows: 4-μm-thick tissue sections were deparaffinized and immersed in 98% formic acid (30 min). After citrated autoclaving (121 °C, 30 min), the endogenous peroxidase activity was blocked (H_2_O_2_ in methanol, 10 min) and slides placed in distilled water overnight at 4 °C. After incubation with 5% normal goat serum (Dako, http://www.dako.com) for 20 min, PrP^sc^ was detected with the monoclonal antibody F99/97.6.1 (2 μg/ml; VMRD, http://www.vmrd.com). Subsequent steps were performed with the peroxidase/DAB Dako Envision detection kit (Dako). PrP^sc^ immunolabeling was scored semi-quantitatively on a scale from 0 (absent) to 3 (severe).

For additional information about Materials and Methods see Protocol S1.

## Supporting Information

Protocol S1 Supplementary Methods (31 KB DOC)

### Accession Numbers

Sequences were translated from the GenBank database (http://www.ncbi.nlm.nih.gov/Genbank) and the accession numbers were caprine (EF140716), human (NM_183079), and ovine (U67922).

## Abbreviations

BSE: bovine spongiform encephalopathy
CS: clinically suspicious
EMP: electrophoretic mobility pattern
FS: fallen stock
GSS: Gerstmann-Sträussler-Scheinker disease
IHC: immunohistochemistry
PrP: prion protein
PrP^c^: host-encoded normal prion protein
PrP^res^: proteinase K–resistant core fragment of PrP^sc^
PrP^sc^: abnormal partially proteinase K–resistant isoform of PrP^c^
RS: regularly slaughtered
SR: small ruminant
TSE: transmissible spongiform encephalopathy
WB: western immunoblot
