# Supplementary material for: Diversity in Neuroanatomical Distribution of Abnormal Prion Protein in Atypical Scrapie
Source: PLoS Pathog. 2007 Jun 8;3(6):e82. doi: 10.1371/journal.ppat.0030082 (PMC1891327; doi:10.1371/journal.ppat.0030082)
Supplement: Supplementary file 1 [file ppat.0030082.sd001.doc]

# Supplementary methods

# TSE cases and reference material. From regularly slaughtered SR the brainstem, sometimes also with remnants of cerebellum and from fallen stock animals the whole brain as well as retropharyngeal lymph nodes and tonsils were available for further analysis.

Reference material from Swiss classical scrapie cases originated from two sheep (PrP genotype ARQ/VRQ) inoculated by the *i.v.* route with CNS material from a clinically scrapie affected ram (PrP genotype VRQ/VRQ) diagnosed in Switzerland in 1999. The inoculum consisted of 2 ml 10% (w/v) brain tissue homogenate in a 320 mM sucrose solution, diluted to 20 ml in 0.9% NaCl. Prior to inoculation the homogenate was heated twice at 80°C for 10 min to inactivate viral and bacterial contaminants. After the onset of scrapie specific clinical signs, the animals were euthanized. Immunohistochemistry (IHC) and discriminatory Western immunoblot (WB) confirmed that both animals were affected by classical scrapie. One of the British reference classical scrapie cases had the genotypes ARH/VRQ and four the genotype ARQ/VRQ.

**TSE screening tests**. Check Western SR (referred to as screening test A): briefly, 100 μl of a 10% (w/v) brainstem tissue homogenate in a test specific homogenisation buffer was incubated for 30 min at 37°C with proteinase K in duplicates. The reaction was stopped with a proteinase inhibitor and SDS sample buffer was added to a final volume of 200 μl. The samples were then denatured for 5 min at 96°C and 10 μl were loaded on 12% NuPAGE gels (Invitrogen) and separated by SDS PAGE. After electrophoresis the proteins were transferred to PVDF membranes (Millipore) and incubated for 60 min with a cocktail of two PrP specific primary antibodies. Antigen-antibody complexes were visualized with an alkaline phosphatase-labelled secondary antibody, followed by addition of CDP substrate (Applied Biosystems) and exposure of the membrane to a photographic film (Amersham Biosciences).

TeSeE Sheep/Goat (referred to as screening test B): 250 µl of a 20% (w/v) tissue homogenate were filtered through a calibration syringe (Bio-Rad) and incubated 10 min at 37°C with a buffer containing proteinase K. Remaining proteins were recovered as a pellet after protein precipitation and centrifugation for 5 min at 20,000 g. Supernatants were discarded, pellets dried and resuspended. After denaturation for 5 min at 100°C, PrP^res^ was detected in a Sandwich ELISA procedure.

**Refined Western immunoblotting.** PrP^res^-concentration was performed according to the protocol of the TeSeE Sheep/Goat Western Blot (Bio-Rad). Briefly 20% (w/v) tissue homogenates were prepared in a Ribolyser (FastPrep® - Qbiogene). Of these, 500 µl were filtered through a calibration syringe and incubated 10 min at 37°C with a buffer containing proteinase K. Remaining proteins were recovered as a pellet after protein precipitation and centrifugation for 7 min at 15,000 g, resolubilized in Laemmli sample buffer (62.5 mM Tris-HCl pH 6.8, 2% (w/v) SDS, 25% (w/v) glycerol and 0.01% (w/v) bromophenol blue) and boiled for 5 min at 100°C. After a second centrifugation for 15 min at 15,000 g the supernatant was transferred into a new tube and the pellet discarded. 15 μl of the supernatants were loaded on 15% SDS hand cast polyacrylamide gels. SeeBlue Pre-Stained Standard (Invitrogen) served as molecular mass marker. After electrophoresis, the proteins were transferred to 0.45 μm supported nitrocellulose (Bio-Rad). The membranes were immersed in phosphate buffered saline (PBS), washed with distilled water and then blocked for 30 min with blocking buffer (Prionics). After incubation with the monoclonal antibody (MAb) P4 (R-Biopharm, 400 ng/ml) for 30 min, the membranes were washed three times in PBS containing 0.1% Tween 20 and then incubated for 20 min in the goat anti-mouse antibody IRDye 800 (Rockland, 200 ng/ml). The blots were scanned with the Odyssey infrared imager (LI-COR Biosciences) at a wavelength of 800 nm and a resolution of 169 μm. Protein-bands were identified, quantified and their molecular mass estimated with the Odyssey analysis software v. 2.1 (LI-COR Biosciences).

**Genotyping.** Genomic DNA was extracted from brain tissue using the Blood & Cell Culture DNA midi kit (QIAGEN) and the complete 771 nucleotide ovine PrP coding region was PCR amplified using primers F 5’-TTT TAC GTG GGC ATT TGA TG-3’ and R 5’-ACA GGG CTG CAG GTA GAC AC-3’ and analyzed by direct sequencing of PCR products with the ABI BigDye Terminator Sequencing Kit 3.1 (Applied Biosystems). The sequencing reactions were ethanol purified and analyzed on an ABI 3730 capillary sequencer (Applied Biosystems). Ovine sequences were compared on the basis of GenBank accession number U67922. Goats were genotyped as described previously [1].

# Reference

1.	Seuberlich T, Botteron C, Benestad SL, Brünisholz H, Wyss R, Kihm U, Schwermer H, Friess M, Nicolier A, Heim D, Zurbriggen A (2007) Atypical scrapie in a Swiss goat and implications for transmissible spongiform encephalopathy surveillance. J Vet Diagn Invest 19: 2-8.
